# African swine fever in the era of global expansion: Epidemiology, transmission dynamics, viral evolution, and One Health control strategies

**DOI:** 10.14202/vetworld.2026.3351-3371

**Published:** 2026-08-04

**Authors:** Jia Bie

**Affiliations:** 1 College of Agronomy and Life Sciences, Kunming University, Kunming, China.

**Keywords:** African swine fever, biosecurity, disease control, epidemiology, One Health, surveillance, transmission dynamics, viral evolution

## INTRODUCTION

African swine fever (ASF), caused by the African swine fever virus (ASFV), is a highly contagious and often fatal hemorrhagic disease affecting domestic pigs and wild boars [1, 2]. ASFV belongs to the family *Asfarviridae*. It is a large, complex, enveloped double-stranded DNA virus characterized by significant environmental resistance and genetic diversity [3]. Since its first identification in 1921, ASF has transcended its original geographic boundaries through multiple intercontinental spread events. Notably, genotype II strains have rapidly disseminated across Europe, Asia, and more recently the Caribbean since 2007 [3]. The disease has been reported in numerous countries across Africa, Europe, and Asia, placing over half of the global pig population at risk and causing substantial economic losses [4, 5].

The economic impact of ASF is profound, threatening the sustainability of the global swine industry, disrupting international trade, and jeopardizing food security and social stability, particularly in regions where pork is a dietary staple [6, 7]. In China, approximately 1.2 million pigs were culled in 2019, resulting in a sharp 27.5% drop in pig inventory [8]. ASF has reduced pig herds by over 40% in some parts of Asia (Food and Agriculture Organization [FAO], 2026). In Spain alone, export losses caused by the outbreak reached several hundred million euros in 2025, while smallholder farmers in the Philippines suffered an average direct economic loss of about US$737 per household (Tridge News) [9]. In Kenya, annual ASF losses amount to approximately US$7 million (FAO, 2025). Data from a 13 January 2026 press conference by Unió de Pagesos, the Catalan farmers’ union, estimated that ASF has cost the region’s pig industry approximately €63 million, with a 17% decline in turnover from November to December, underscoring the outbreak’s severe impact (Pig World News). The United States Department of Agriculture (USDA) projects a 9.2% year-on-year decrease in EU pork exports in 2026. These disruptions highlight how a single ASF detection can generate global market ripple effects, harming producers well beyond the initial outbreak site (USDA, Foreign Agricultural Service). Consequently, ASF is listed as a notifiable disease by the World Organization for Animal Health (WOAH) [10].

For the period from 1 January 2022 to 28 February 2026, ASF was reported in 74 countries across five world regions, affecting 1,152,369 domestic pigs and 48,613 wild boars, with total animal losses reaching 2,571,160. Asia and Europe remained the most affected regions (WOAH, Situation Report 75). In the Caribbean, outbreaks in the Dominican Republic and Haiti, first detected in 2021, continue to pose a persistent threat to the Americas; however, to date, no further spread to new countries on the American mainland has been reported. From January 2023 to March 2024, the Dominican Republic received 9,422 surveillance reports, of which 2.45% tested positive, indicating a transition from acute outbreaks to endemicity [11]. Meanwhile, Oceania reported no new events in 2025, but ongoing long-distance “jump” transmissions globally underscore that the epidemic remains uncontrolled. Papua New Guinea experienced a significant ASF event before March 2026 (and was therefore not included in the statistics). The country reported the first occurrence of the disease in a new region, East Sepik, with the outbreak start date retrospectively set to December 2025. The region practices free-range farming with frequent interaction with a large wild boar population, and its geographical isolation makes the Sepik River the main transport route (WOAH, Situation Report 75).

While the overall number of ASF outbreaks in domestic pigs continued to decline globally in late 2025, wild boar cases remained persistently high with considerable regional heterogeneity (WOAH, Situation Reports). In Germany, North Rhine-Westphalia had exceeded 500 infected wild boars by March 2026, and a single case reappeared in Saxony in April 2026, highlighting the risk of reintroduction even in low-density settings (Food and AgriBusiness). In Asia, South Korea experienced a surge (24 farm outbreaks and 1,022 wild boar positives by March 2026) (Food and AgriBusiness), while the Philippines saw a dramatic drop (only 19 barangays active by February 2026) (Philippine news agency). Since the detection in November 2025 in Catalonia (Spain), ASF has been confirmed exclusively in wild boar populations, with no cases detected in domestic pigs. As of mid-April 2026, 44 outbreaks involving 252 ASF-positive wild boars have been reported, all confined to restricted areas. These data show that, despite cautious optimism in some regions, the global trend of declining domestic outbreaks alongside persistent or rising wild boar cases is underpinned by strong country-level heterogeneity, which must be accounted for in ongoing surveillance and risk-based strategies. Like highly pathogenic avian influenza (HPAI), ASF faces the challenge of a wildlife reservoir [12]. ASF shares key similarities with the COVID-19 pandemic, including trade restrictions and the role of human mobility in long-distance viral spread. However, unlike COVID-19, ASFV does not infect humans, a fact that may paradoxically lower political urgency and public attention, impeding resource allocation for control efforts. In May 2025, WOAH issued the first international standards for ASF vaccine production, followed later by expert guidelines on field evaluation and post-vaccination monitoring. Nevertheless, as of June 2026, no country/territory has formally notified WOAH of large-scale vaccination in areas around confirmed outbreaks (WOAH). The virus’s genetic complexity and ability to evade host immune responses complicate vaccine development [5, 13]. Therefore, strict biosecurity measures remain the cornerstone of ASF prevention and control.

Given the continuous global spread of ASF, there is a pressing need to synthesize existing knowledge on its epidemiology. This review aims to comprehensively delineate the global epidemiological characteristics of ASF, critically analyze the key drivers of its transmission and persistence, and evaluate the effectiveness of current control measures. The findings are intended to inform the development of predictive surveillance systems and provide evidence-based guidance for designing effective containment and mitigation strategies.

Although numerous reviews have addressed specific aspects of ASF, significant gaps persist in integrating recent global data. These include the post-2025 divergence between declining domestic pig outbreaks and rising wild boar cases, the emergence and field impact of genotype I/II recombinants and vaccine-like strains, practical challenges of vaccination in smallholder systems, and the translation of WOAH standards into diverse production contexts across continents. Limited synthesis exists on how climate factors, human mobility, illegal vaccine use, and viral evolution interact to shape long-term control outcomes.

This review aims to (1) synthesize the global spatiotemporal epidemiology of ASF from 2007 to June 2026 with updated post-2025 data, (2) critically analyze transmission dynamics and drivers with emphasis on wildlife reservoirs and human-mediated pathways, (3) evaluate recent viral evolutionary trends including recombinants and vaccine-associated variants, and (4) assess current control strategies within a One Health framework while identifying evidence-based priorities for surveillance, biosecurity, and adaptive vaccination programs.

## REVIEW METHODOLOGY

### Literature search

A literature search was conducted in June 2026 across three databases (PubMed, Web of Science, and CAB Abstracts) using the Boolean search string: ("African swine fever" OR ASF OR ASFV) AND (epidemiology OR transmission OR genotype OR control OR vaccine). The search covered January 2007 to June 2026. Additional sources included WOAH’s World Animal Health Information System (WAHIS) database, European Food Safety Authority (EFSA) reports, FAO publications, official news reports from various regions, and reference lists of key papers.

### Inclusion criteria

Studies on ASFV genotype II or global/pandemic aspects; peer-reviewed original research, reviews, or official reports; published in English; published after 2007 (foundational work excepted).

### Exclusion criteria

Purely laboratory studies lacking epidemiological context; non-peer-reviewed sources.

### Study selection

Study selection was performed by a single author. Titles and abstracts were screened first, followed by full-text assessment. After the initial screening, a total of 191 articles were downloaded and roughly categorized as follows: 55 on fundamental ASF research, 44 on epidemiology, 36 on vaccines, 21 on viral recombination, regional epidemiological characteristics, and farming systems, and 35 on official documents. Some references cited in the text were news reports and were read directly on their respective websites rather than downloaded for full-text review. Data extraction followed a thematic synthesis approach, focusing on virus evolution, transmission pathways, control measures, and vaccine development. No formal quality assessment was performed due to the narrative nature of this review; however, priority was given to high-impact journals, WOAH/FAO reports, and whole-genome sequencing studies.

### Limitations

Single-author screening may introduce selection bias. Chinese- and Russian-language literature was not cited in this review; although data on China and Russia were supported by English-language sources, the restriction to English may have resulted in the omission of important non-English studies. The narrative format precludes quantitative synthesis. The rapidly evolving ASF landscape means post-cutoff developments may not be fully captured.

## SPATIOTEMPORAL CHARACTERISTICS OF GLOBAL ASF DISTRIBUTION

### Historical origin and global dissemination pathway

ASF is believed to have originated in a natural enzootic cycle involving warthogs and soft ticks in sub-Saharan Africa, where it was first identified in Kenya in 1921. For decades, the disease remained largely confined to this region [14]. The first transcontinental leap occurred in 1957 to Portugal, linked to contaminated food waste from aircraft [15]. Subsequent introductions were reported in other European countries and Latin America [16]. While control measures led to its eradication from most of Europe (except Sardinia, Italy) by 1995, a pivotal turning point in the modern global spread was the introduction of a genotype II strain from southeastern Africa to Georgia in 2007 [15].

**Figure 1:**
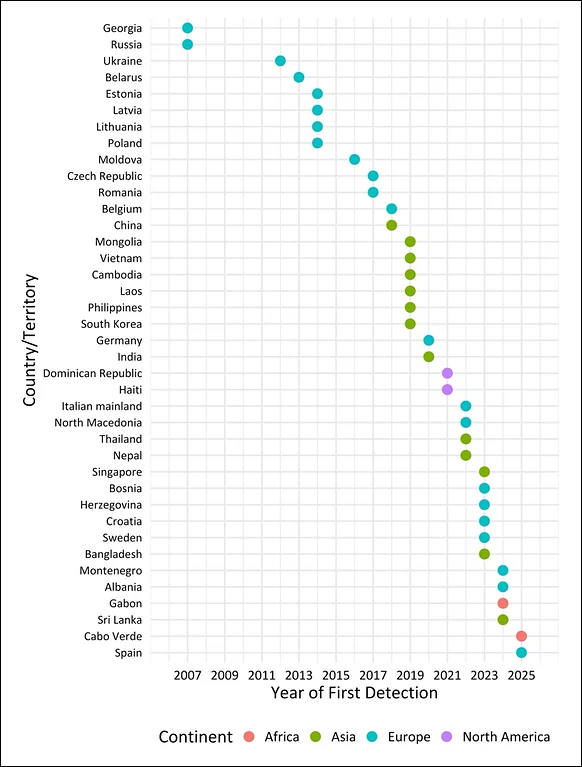
Spatiotemporal timeline of global ASFV genotype II spread.

From Georgia (Figure 1), ASF rapidly disseminated across Eastern Europe, reaching Russia (2007), Ukraine (2012), Belarus (2013), and the European Union (Estonia, Latvia, Lithuania, Poland in 2014). It further spread to Moldova (2016), the Czech Republic, and Romania (2017). The virus was later detected in wild boar in Belgium (2018) and Germany (2020), marking its establishment in Western Europe [15, 17–20]. In 2018, ASF entered Asia, with China reporting its first outbreak. The virus then spread explosively across the continent, affecting Mongolia, Vietnam, Cambodia, Laos, the Philippines, South Korea, India, and others within a short period (2019-2020) [21–24]. In July 2021, the disease reappeared in the Americas after nearly 40 years, with first introductions in the Dominican Republic and later Haiti. In January 2022, ASF genotype II was detected on the Italian mainland following a similar 40-year absence, while North Macedonia and Thailand also reported their first occurrences that month. Nepal followed with its first report in March 2022. Throughout 2023, first-time detections were recorded in Singapore (February), Bosnia and Herzegovina (June), Croatia (June), Sweden (August), and Bangladesh (November). The spread continued in 2024, with Montenegro (January), Albania (February), Gabon (June), and Sri Lanka (December) each confirming the disease for the first-time. In March 2025, Cabo Verde reported an ASF outbreak to WOAH. On 28 November 2025, Spain confirmed its first ASF case in wild boar in Catalonia, marking the country’s first outbreak since 1994 (WOAH; USDA). Historical and recent transboundary spread is primarily attributed to the movement of contaminated pork products, illegal transport of infected animals, and, in Europe, the natural movement of infected wild boar [25–27]. The Caribbean region (Dominican Republic, Haiti) remains a persistent “hotspot” for ASF in the Americas, with ongoing virus circulation. Since its first outbreak in 2021, the Dominican Republic has entered an endemic phase, and during the first half of 2025, the number of reported outbreaks continued to rise, spreading across multiple areas of the country. In response, the Dominican government, in coordination with the FAO and the USDA, launched a national pig biosecurity program in 2023. This comprehensive strategy aims to strengthen ASF prevention and control across all segments of the production chain, with a particular emphasis on animal transport management (FAO News). However, the risk of ASF incursion into North America remains high due to frequent human movement (particularly tourism) with these countries (USDA News).

### Analysis of current regional epidemiological patterns

The global ASF landscape is now characterized by distinct regional patterns (Table 1). In Europe, the virus is self-sustaining in wild boar populations across many countries, creating a resilient reservoir that complicates eradication efforts [28]. Countries like Poland show evidence of the epidemic transitioning toward endemicity [29]. While the Czech Republic and Belgium successfully eliminated localized outbreaks, Germany has experienced multiple introductions and a recent spread to its western regions, highlighting persistent challenges [20]. The spread of ASFV in Europe is characterized by a combination of long-distance jumps and regional diffusion, consistent with Poland’s role as a genetic center. Strains from domestic pigs and wild boar are genetically indistinguishable, which may reflect low diversity and sampling gaps rather than frequent cross-species transmission [30]. In November 2025, Spain detected ASF in two wild boars in the province of Barcelona, marking its first recurrence in 31 years, with a jump distance of approximately 599 km. Moreover, the Animal and Plant Health Agency (APHA) report noted that since the first detection in June 2025, ASF cases have continued to occur in wild boar in North Rhine-Westphalia, marking a small-scale westward expansion (Pig World News). After five and a half years of control efforts, Saxony declared eradication in February 2026, but less than two months later (in early April 2026), ASFV was again detected in a wild boar carcass in the Görlitz district, underscoring the importance of continued surveillance after eradication. Wild boar cases across Europe have been rising since autumn 2025, with 903 cases reported in January 2026, for example, underscoring the persistent wildlife reservoir (WOAH) [10].

**Table 1:** Summary of global African swine fever outbreaks by region and host type, January 2022 – February 2026.

**Region**	**Host type**	**Number of outbreaks**	**Number of cases**	**Domestic pig losses**
Africa	Domestic pig	1,160	141,672	137,586
Africa	Wild boar	6	0	—
Americas	Domestic pig	65	467	9,412
Americas	Wild boar	0	0	—
Asia and Pacific	Domestic pig	7,246	310,633	724,615
Asia and Pacific	Wild boar	110	568	—
Europe	Domestic pig	5,408	699,597	1,699,547
Europe	Wild boar	30,314	48,045	—
Global Total	Domestic pig	13,879	1,152,369	2,571,160
Global Total	Wild boar	30,430	48,613	—

Data in this table are extracted from WOAH ASF Situation Report No. 75, containing cumulative records up to 28 February 2026. Domestic pig losses are the number of pigs that died or were culled within outbreak sites, excluding animals culled in surrounding zones for disease control. The Asia and Pacific region follows WOAH official geographical grouping, covering all Asian and Oceanian countries and territories. Separate statistics for wild boar losses are not provided in WOAH reports, indicated by the symbol “—”.

The epidemiological situation in Asia presents a contrasting picture, characterized by remarkably rapid spread compared to Europe, likely attributable to high swine density, extensive trade networks, and widely varying biosecurity levels across production systems [21]. Molecular epidemiological studies suggest multiple independent introductions of ASFV into China, with the virus subsequently spreading predominantly southward from initial outbreak clusters in the northeastern part of the country [31, 32]. The emergence of novel variants and recombinant strains represents the most serious challenge in Asia. China has reported recombinant ASFV strains of genotypes I and II [33]. Vietnam, the Russian Federation, Thailand, and other countries have continuously reported new variant strains [34–37]. In July 2025, Cambodia experienced an ASF jump of approximately 146 km from the nearest reported outbreak, demonstrating the risk of long-distance spread mediated by human activities. In November 2025, Bhutan reported a recurrence of ASF. After seven months without any cases, a further recurrence was confirmed in January 2026 in a small backyard herd in the southwestern Chukha district, within 10 kilometers of the Indian border, according to a WOAH notification. Swill feeding has been cited as a contributing factor (Feed Business News). Reports in early 2026 indicate that South Korea continues to experience new outbreaks, with ongoing active cases in wild boar populations (WOAH). In contrast, the Philippines saw a gradual decline in ASF outbreaks throughout 2025, with a rapid improvement in early 2026, indicating that the epidemic has been effectively controlled there.

As the origin and global epicenter of genetic diversity for ASFV, Africa harbors a unique sylvatic cycle and 24 genotypes. This distinctive sylvatic cycle makes Africa a natural gene pool for ASFV and poses a continuous risk of spillover into domestic pig populations [38, 39]. Based on the *p72* (*B646L*) gene, 24 ASFV genotypes have been identified, all of which are distributed across Africa. East, Central, and Southern Africa are the cradle of ASFV, harboring all known *p72* genotypes globally. However, of these 24 genotypes, only five (I, II, VIII, IX, and X) have a wide field distribution in domestic pigs. Among the genotypes that appear strongly adapted to domestic pigs, two have spread beyond the African continent and have become the focus of ASF vaccine development [40, 41]. In recent years, the first co-circulation of genotypes I and II has emerged in West Africa [42], significantly increasing the risk of recombination and posing a new threat to the regional pig industry. At the same time, widespread under-reporting of surveillance and weak biosecurity among smallholders across the continent further complicate ASF control. More critically, currently available live-attenuated vaccines (LAVs) targeting genotype II lack effective cross-protection against other genotypes circulating in Africa, particularly genotype IX, presenting a new scientific challenge for future vaccine strategies in Africa [43]. The Fifth Standing Group of Experts (SGE) Meeting of the WOAH Africa Region has explicitly highlighted the urgent need to develop vaccines targeting locally circulating genotypes in Africa and to strengthen regional surveillance networks.

ASF continues to pose a severe challenge in Africa. In June 2025, Mali reported its first outbreak since June 2023. Angola reported the first occurrence of ASF in a new region in December 2025. Cabo Verde reported the first occurrence of ASF in a new region in February 2026 (WOAH). Furthermore, the persistent co-circulation of ASFV genotypes I and II continues in Nigeria, Burkina Faso, Côte d’Ivoire, and Mali, significantly increasing the risk of recombination [42]. The pig production system in Africa is dominated by smallholder free-range farming, characterized by low biosecurity levels and weak veterinary oversight, and outbreaks often go unreported in a timely manner. In response to these challenges, the Global African Swine Fever Research Alliance (GARA) Africa chapter was established in recent years to enhance ASF diagnostic capacity and regional cooperation by strengthening collaborative networks among African veterinary diagnostic laboratories [44, 45]. In Africa, smallholder pig systems have long been associated with low levels of biosecurity, where poverty and structural constraints severely limit the effective implementation of biosecurity measures [46]. Taking Gauteng Province of South Africa as an example, ASF outbreaks in 2012 and during 2019–2022 exposed significant biosecurity shortcomings among smallholders. A systematic assessment found that key risk factors included a lack of isolation facilities, improper disposal of dead pigs, and limited awareness among farmers regarding ASFV transmission [47]. Studies in countries such as Uganda have also shown that while implementing biosecurity interventions alone can reduce ASF outbreaks, it can also reduce profit margins for farmers. Therefore, without corresponding economic incentives, such as cost-sharing mechanisms or subsidies, smallholders are unlikely to adopt necessary biosecurity measures voluntarily [48].

### Spatiotemporal dynamic modeling

Spatiotemporal models have revealed key patterns in ASF spread that are critical for informing control efforts. Analysis of nine domestic pig ASF outbreaks in Germany between 2021 and 2024 confirmed that wild boar was the main source of infection. In Lower Saxony, a novel mutant strain was identified in 2022; in Brandenburg, the genetic sequences of domestic pig outbreak strains were highly consistent with those of surrounding wild boar isolates, supporting wild boar as the source of the virus [49]. Cross-infection between wildlife and domestic pigs further complicates ASF control. A multi-host transmission model has quantitatively demonstrated that cross-species transmission is a key driver of ASF epidemics. The model estimates that approximately 60% of pig cases originate from other infected pigs, while 27% originate from infected wild boar populations. For wild boar infections, about 39% are derived from infected pigs and 61% from other infected wild boars [50].

In temperate regions of Europe and Asia, ASF outbreaks in both wild boar and domestic pigs exhibit distinct seasonal peaks, often occurring in summer and autumn/winter. These patterns are closely linked to wild boar ecology, such as reproduction and movement, as well as human activities including harvesting and hunting [28, 51]. Ecological drivers of ASF include enhanced viral survival at low temperatures, winter boar aggregation at feeding sites, seasonal vector activity, and periodic acorn mast years that boost boar density and contact rates. Similarly, researchers suggest that increased agricultural activities in summer, along with the greater frequency and abundance of arthropod vectors during warmer months, may also contribute to the higher incidence of ASF observed in summer [52]. Global spatiotemporal dynamic analyses of ASF have confirmed significant statistical associations between climatic factors (temperature, precipitation) and transmission risk, underscoring the need to integrate climate change into the risk assessment framework of long-term ASF control strategies [53]. Their complex interactions may drive abnormally high population densities and elevate the risk of outbreaks [54, 55]. The ASF Challenge Project has shown that accounting for livestock-wildlife interactions is essential for improving emerging disease control and strengthening scientific preparedness for future ASF outbreaks [56].

By contrast, seasonality is considerably less pronounced in tropical Asia. Analyses of transmission dynamics in Ukraine estimated an average spread rate of 5.2 km per day, with transmission originating from wild boar progressing more slowly than that originating from domestic pig sources [57]. In Asia, a clear southward propagation from Northeastern China to Southeast Asia was observed [32]. The spatiotemporal pattern in Vietnam evolved from a directed, clustered spread in the early phase to more random, smaller-scale outbreaks later [58]. Spatial clustering patterns further illuminate underlying transmission processes: the presence of multiple, distinct spatial clusters in Germany suggests repeated introductions of the virus, whereas the identification of single clusters in the Czech Republic and Belgium points to localized, human-mediated introductions that were subsequently successfully contained [59]. Across these diverse spatiotemporal patterns, a consistent theme emerges: the predominant role of human activities in facilitating long-distance viral spread. Future spatiotemporal models should therefore integrate wildlife density, human mobility patterns, and genomic sequencing data to improve the prediction of ASF transmission risk.

Most existing models rely on retrospective outbreak data to estimate parameters such as R₀ and transmission rates, whereas human mobility data (e.g., livestock transport routes, market networks, and international pork product flows) and genomic data remain underutilized in ASF transmission modeling [42, 50, 60, 61]. To address the complex transmission dynamics of ASF, an AI-augmented predictive dashboard can be developed that integrates real-time price signals, genomic variants, and human mobility data. This dashboard would systematically fuse three data layers: real-time market data (e.g., pork wholesale prices, feed costs, supply–demand indices) as early warning signals of socio-economic drivers; genomic variants (e.g., deletion patterns, recombination events) to enable fine-scale tracking of viral lineage evolution; and real-time human mobility data (e.g., livestock transport routes, wild boar density, border inspection records) to construct dynamic mapping of transmission networks. By combining traditional epidemiological models with machine learning algorithms, the system aims to deliver real-time risk updates and iterative improvements in predictive accuracy. The EFSA has already used reliable national and regional wild boar density data, provided by European experts, to calibrate abundance models based on hunting statistics. This unified, high-resolution European wild boar density map can be directly applied to epidemiological and risk analysis models (Farm Progress News, 2026). Given the rapid advances in AI, such predictive support systems are now ready for practical deployment. The proposed AI-augmented predictive dashboard can build on these foundations, further enhancing predictive tools to achieve higher performance.

## ANALYSIS OF EPIDEMIOLOGICAL LINKS AND TRANSMISSION DYNAMICS

### Diversity of infection sources

ASFV is maintained through distinct epidemiological cycles. The ancient sylvatic cycle involves warthogs and soft ticks (*Ornithodoros* spp.) in sub-Saharan Africa [62–65]. The domestic cycle, which can operate independently of ticks, is sustained through direct and indirect contact between infected and susceptible pigs [64]. The emergence of ASFV in soft ticks beyond traditional endemic areas indicates a potential geographical expansion of the sylvatic cycle, posing an additional challenge in Africa [16].

In Europe, wild boars have become a critical reservoir, establishing a self-sustaining transmission cycle without the need for ticks or domestic pigs. Infected wild boar and their carcasses act as potent viral reservoirs, facilitating long-term environmental persistence and spillover to domestic herds [28]. Spatiotemporal analyses confirm a significant association between wild boar activity hotspots and ASF outbreaks in domestic farms [66]. Within the domestic pig sector, clinically ill and dead animals are primary sources. The movement of live pigs, particularly through high-centrality nodes like genetic nucleus units and multiplier farms, plays a crucial role in network-based spread [64, 67, 68]. The role of chronically infected “carrier” pigs remains debated but may contribute to persistent transmission in some contexts [38].

Recent studies have confirmed that live-attenuated vaccine-related ASFV strains are circulating persistently in domestic pig populations in Vietnam, with significantly increased genetic diversity. Following vaccine commercialization, viral micro-variants have emerged that are both similar to vaccine strains and represent novel wild-type forms [69]. It is hypothesized that high restocking rates and inadequate biosecurity measures were the main pathways for the introduction of the strain [70]. In addition, vaccine-like ASFV strains bearing characteristics of laboratory-attenuated viruses have been detected in domestic pigs in Thailand (2024–2025) [35]. Although subtle, these meaningful genetic changes indicate that ASFV may be undergoing rapid evolution through large-scale vaccine-driven immunity and multiple sources of introduction during re-emerging transmission, potentially allowing the virus to evade host immune recognition [69]. These findings warn that the use of genetically engineered vaccines, especially under conditions of improper handling or leakage, may lead to the emergence of novel variants, posing new threats to regions without a history of vaccination or those that have achieved eradication. They underscore the critical need for stringent biosecurity measures and enhanced surveillance.

### Complexity of transmission routes

Direct contact between infected and susceptible pigs is an efficient route for intra-herd spread. The virus is present in blood, secretions, and excretions [71, 72]. Indirect transmission is a major driver of long-distance spread. The virus’s remarkable environmental stability allows it to persist for extended periods in contaminated feed ingredients, on vehicles, equipment, and clothing [15, 73]. Contaminated pork products are a key factor in transboundary spread. ASFV can survive for months in frozen meat and certain processed products like salami, making swill feeding a high-risk practice [74].

While *Ornithodoros* ticks are biological vectors in Africa and parts of Europe, their role is limited in Asia [75]. Mechanical transmission by other arthropods, such as stable flies (*Stomoxys calcitrans*), has been demonstrated experimentally [76, 77]. Although the stable fly has been repeatedly demonstrated to mechanically carry ASFV for up to 48 hours under laboratory conditions, there is no conclusive evidence that insects act as regular vectors of ASF. Nevertheless, indirect evidence, such as experimental transmission and field detection of viral DNA, supports their potential role [78]. An intriguing novel finding suggests certain freshwater snails may act as atypical environmental hosts, potentially contributing to ASFV persistence and spread in aquatic environments [79]. However, evidence for snails as potential “biological reservoir hosts” is currently confined to laboratory conditions, and their epidemiological relevance requires field verification. The actual transmission pathway, dose, and efficiency from snails to susceptible pigs remain unconfirmed, so their role remains theoretical at this stage.

Human activities are the predominant driver of long-distance transmission. The illegal movement of pigs and pork products, combined with inadequate biosecurity practices (e.g., swill feeding, ineffective decontamination of vehicles), facilitates viral introduction into new regions [80]. In Poland, epidemiological sequences where domestic pig outbreaks preceded wild boar cases strongly point to human-mediated spread [29]. The predominance of human-mediated transmission underscores the critical importance of biosecurity and regulation.

### Susceptible animals and influential management factors

Domestic pigs and European wild boar are highly susceptible to ASFV infection. While African wild suids like warthogs often show tolerance, domestic pigs typically experience high mortality rates, particularly with virulent strains [67]. Farm management and biosecurity levels are the primary determinants of outbreak risk and scale. Small-scale or backyard farms with low biosecurity are disproportionately affected [21]. In these systems, swill feeding is a major transmission factor. In many newly affected Asian regions, including the Philippines, Cambodia, and Lao PDR, smallholder farmers face significant constraints in biosecurity knowledge, infrastructure, and financial capacity [81, 82]. In smallholder systems in Africa, a survey in Tanzania found that 80% of farms fed untreated swill [83]. In a study from the Philippines, approximately 30% of interviewed farmers continued this high-risk practice (at the 3rd International Online Conference on Agriculture, session on Farm Animal Production). A modeling study in China quantitatively assessed the impact of swill feeding and found that it significantly accelerated the transmission of ASF. Compared with scenarios without swill feeding, swill feeding could result in a fivefold higher frequency of outbreak peaks and a sevenfold increase in total pig mortality [84]. Reports from Bhutan indicate that behavioral change is extremely difficult for farmers, as many are reluctant to invest in biosecurity and remain committed to traditional farming practices. As a result, the virus persists in free-range and backyard production systems, posing an ongoing obstacle to regional eradication. In contrast, for large-scale commercial farms, the primary risks often stem from the mechanical introduction of the virus via contaminated vehicles and personnel [74]. The high density of smallholdings in Asia, coupled with frequent animal movements, contributed to the explosive spread of ASF in the region [21].

Based on the evidence presented, within the Transmission Risk Hierarchy for ASF, high-risk factors include swill feeding and illegal trade of pork products; medium-risk factors involve mechanical carriage by humans and vehicles; low-risk factors include blood-feeding stable flies and other mechanical vectors. Freshwater snails and other gastropods represent a potential risk that requires further validation.

In response to biosecurity challenges among smallholders, FAO launched the Community African Swine Fever Biosecurity Intervention (CABI) program, which promotes practical biosecurity measures through training, facility support, and ongoing monitoring to strengthen resilience and food security in vulnerable communities (FAO News, 2025). In July 2025, China’s Animal Disease Prevention and Control Center issued the “Eight Dos and Eight Don'ts” for ASF control on small- and medium-scale pig farms, guiding improved husbandry, disease prevention, quarantine supervision, and carcass disposal. Meanwhile, although culling remains a key ASF control measure, the Philippine experience demonstrates that its implementation in low- and middle-income countries is hindered by financial, human resource, and farmer acceptance challenges, highlighting the need for complementary economic incentives [85].

**The resilience of ASF within its endemic range varies by region:** Europe’s resilience hinges on wild boar management and long-term strategies, while Asia and Africa rely on smallholder systems, biosecurity and vaccination policies, and, in Africa, the continent’s status as the virus’s origin. Despite decades of research and growing vaccine deployment, ASF remains stubbornly difficult to eradicate. The key to intervention lies in disrupting any one of the three elements of the pandemic cycle, eliminating the wildlife reservoir, blocking human-mediated transmission routes, or staying ahead of viral evolution with adaptive vaccines. In addition to vaccines and biosecurity, developing gene-edited pigs with ASFV resistance or tolerance is becoming a promising long-term complementary strategy. Zheng *et al.* [86] developed a multiplex CRISPR-Cas system targeting the ASFV genome and evaluated its antiviral activity *in vitro* and *in vivo*. The system significantly reduced viral replication *in vitro*, and germline-edited pigs expressing it showed normal growth with sustained guide RNA expression. Although not a substitute for core interventions like vaccines and biosecurity at present, this approach represents an important forward-looking technological reserve [86].

### Virus evolution and genotype distribution

**Figure 2:**
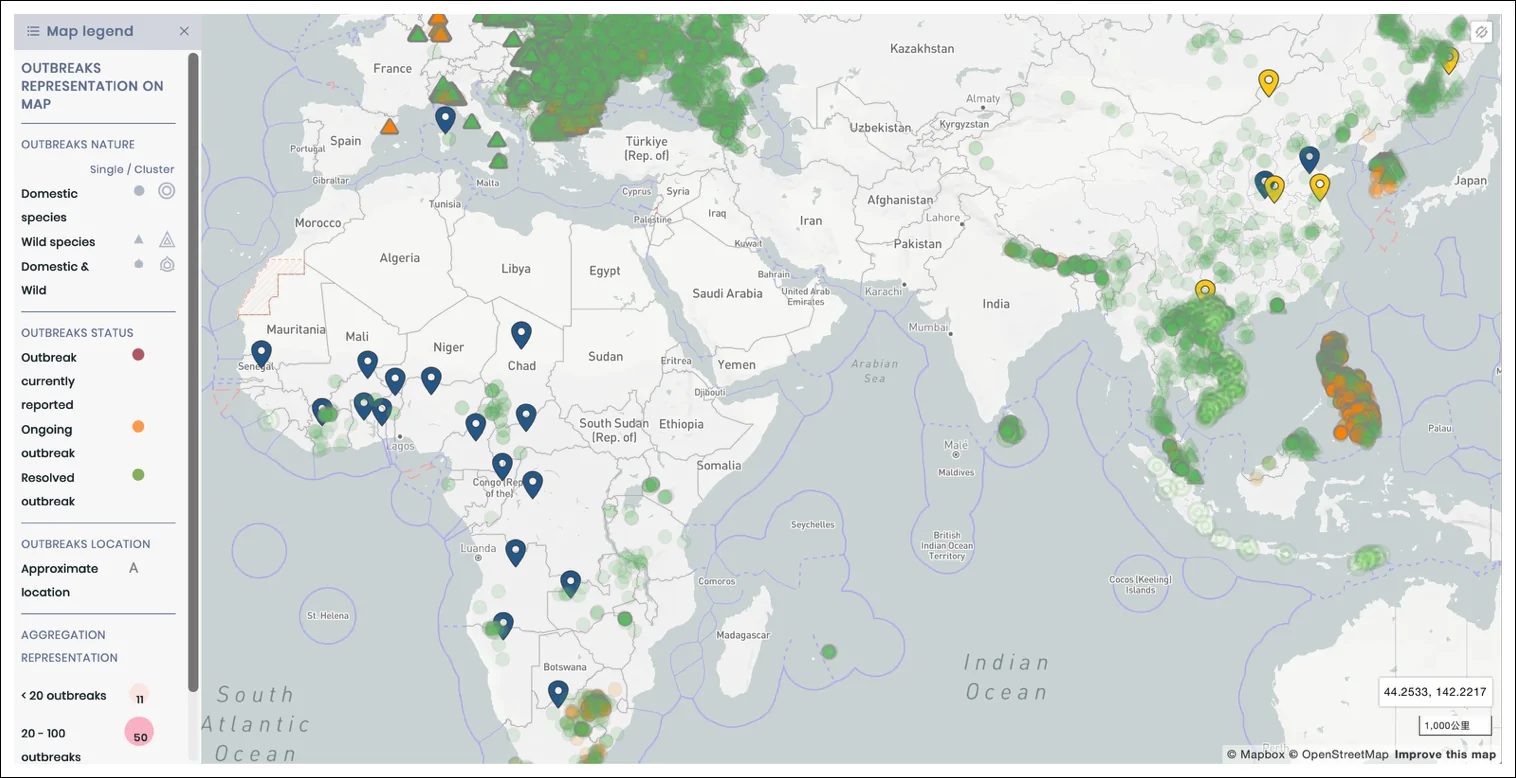
Global distribution of African swine fever virus (ASFV) genotypes and recombination events. The base map was generated using the WOAH interactive map, which shows the geographic background and all ASF outbreaks reported from January 2007 to June 2026 (outbreaks are displayed regardless of genotype). Overlaid symbols indicate the locations of specific genotype events: dark blue icons represent ASFV genotype I detections, and yellow icons represent genotype I/II recombinant strains. Outbreak data were derived from WOAH WAHIS and situation reports, while genotype and recombination event data were compiled from peer-reviewed literature and official notifications as cited in the main text [Source: https://wahis.woah.org/#/home].

**Global distribution patterns of genotypes:** Africa, the origin, hosts all 24 genotypes, with the highest diversity in the Eastern and Southern regions [38]. In contrast, occurrences outside Africa have historically been dominated by two genotypes: the previously widespread genotype I and the contemporary genotype II, which emerged globally after 2007 [3]. The genotype II strain, originating in Eastern Africa, has effectively replaced genotype I as the dominant lineage across Eurasia and globally (Figure 2). This shift is attributed to several key factors: its high virulence causing acute and obvious outbreaks, the immunologically naïve pig populations in new territories, its efficient association with global trade and travel enabling long-distance spread, and its successful establishment in wild boar populations in Europe, creating a persistent reservoir [10, 29].

While genotype II is now pandemic, genotype I persists in certain regions, such as parts of Asia and Africa (FAO and WOAH, 2024; https://rr-africa.woah.org/app/uploads/2025/02/Report-of-the-SGE-ASF-IV-vf-EN.pdf), sometimes co-circulating with genotype II. Sardinia has been a traditional endemic focus of ASFV genotype I since 1978 and is currently in the final stage of eradication [87, 88]. In 2021, two genotype I isolates were recovered from clinically diseased pigs on farms in Shandong and Henan provinces in China (Harbin Veterinary Research Institute [CAAS]). Recent studies have reported, for the first time, the presence of ASFV genotype II in Burkina Faso, Côte d’Ivoire, and Mali, and confirmed the co-circulation of genotypes I and II in Nigeria, Burkina Faso, Côte d’Ivoire, and Mali during 2019–2023 [42]. This coexistence creates a complex epidemiological picture, in which acute outbreaks caused by genotype II coincide with often subclinical or chronic infections driven by less virulent genotype I variants, posing significant challenges for surveillance and control [10].

**Analysis of viral evolution trends:** The ongoing evolution of the pandemic genotype II strain is a subject of intense research. Whole-genome sequencing has revealed the emergence of micro-variants and lineage-specific mutations in different regions. For instance, viruses circulating in Germany and Vietnam have been reported to accumulate more mutations than the original Georgia 2007-like strain, potentially indicating local adaptation or variations in pathogenicity [89, 90]. A significant evolutionary development is the detection of natural recombinant viruses in China. These viruses possess genomes composed of segments from both genotype I and II, demonstrating high lethality and transmissibility. The ASFV isolate from Primorsky Region of Russia shares 99.99% sequence identity with the recombinant isolates previously described in China [91]. In Thailand, a vaccine-like ASFV strain carrying a six-gene deletion was identified in an unvaccinated pig herd and was associated with chronic disease [35]. In 2024, 40% of the strains detected in northern Vietnam were genotype I/II recombinants [92]. Samples collected from unvaccinated pig farms in south-central Vietnam confirmed that vaccine-associated ASFV strains have been persistently circulating in the field. Furthermore, following the large-scale rollout of vaccination, viral genomic diversity increased markedly, with the emergence of both vaccine-like genotypes and novel wild-type micro-variants [69]. Additionally, two ASFV variants with large gene deletions were detected in domestic pig farms in South Korea [93]. These findings indicate that vaccine-like and recombinant strains have emerged in multiple Asian countries and regions. A 2025 study on the Asia-Pacific region performed whole-genome sequencing on 25 ASFV-positive samples and found that all shared >99.8% genetic similarity with the Georgia 2007/1 reference genome. However, clear genetic clustering based on geographic origin was observed, and multiple non-synonymous mutations were identified. Whole-genome sequencing provides a powerful tool for proactive surveillance of novel variants carrying functional mutations in key genes, which may be closely linked to ASFV replication capacity and its ability to adapt to different hosts [89]. Crucially, current genotype II-based vaccine candidates may not confer protection against these recombinant viruses, highlighting their potential for immune evasion and representing a major concern for future control strategies, including vaccination [33]. The emergence of these recombinant viruses poses a formidable challenge to vaccine development, potentially rendering current candidate vaccines obsolete.

### Application of genotyping in epidemiological tracing

Despite being a large DNA virus with a relatively slow mutation rate, ASFV's genetic stability is advantageous for molecular tracing. High-resolution molecular tools, such as whole-genome sequencing and analysis of variable genomic regions, allow for precise outbreak investigation [90, 94]. This genotyping approach has been instrumental in confirming the sources of numerous outbreaks. Molecular analyses consistently linked the initial incursion in Georgia in 2007 to a genotype II strain of Eastern African origin. Similarly, the first detections in China and Italy were genetically traced to Georgia 2007-like viruses, confirming transboundary spread from Eastern Europe [15, 95]. Furthermore, genotyping can distinguish between multiple independent introductions and local spread, as demonstrated in Italy where different genetic clusters indicated several distinct viral introductions [95]. Whole-genome sequencing can reveal micro-variants and geographically characteristic mutations, thereby aiding in the tracing of transmission chains [89]. Furthermore, the IGR-I variants reported in South Korea differ from local wild boar strains, suggesting potential new introductions and further underscoring the critical role of genotyping in epidemiological tracing [60]. The 2026 study further noted that, even with ongoing transmission and micro-variants in the Asia-Pacific, ASFV genomes remain >99.8% identical [89]. WOAH has issued standardized genotyping recommendations: sequencing the 3′ end of the *B646L* (*p72*) gene to differentiate genotypes, and analyzing *B602L* tandem repeats and the I73R-I329L intergenic region to distinguish closely related ASFV subgroups. Regions such as *E183L* (*p54*), *CP204L* (*p30*), and *EP402R* (*CD2v*) are also useful for tracing geographical origin and transmission. To detect genotype I/II recombinants, Chinese researchers developed a triplex quantitative polymerase chain reaction (qPCR) targeting *B646L*, *A151R*, and *MGF360-14L*, achieving a 10 copies/µl detection limit, no cross-reactivity, and a coefficient of variation below 2% [96].

### Evaluation of control measures and associated challenges

The cornerstone of ASF control in non-endemic regions relies on a suite of classical measures. These include the stamping out of infected herds, the imposition of movement restrictions on pigs and pig products, the enhancement of biosecurity protocols at farms and at transport interfaces, and the active management of wild boar populations. In practice, however, the efficacy of any given strategy is ultimately contingent on rigorous implementation and sustained resources, factors that vary considerably across regions and production systems. Biosecurity is the most critical and effective line of defense for individual farms. This involves implementing closed herd management, strict controls on vehicle and personnel entry (including decontamination procedures), and prohibiting the feeding of swill or food waste [32, 74]. Innovative tools, such as Germany’s online “ASF Risk Traffic Light,” have been developed to help farmers self-assess and strengthen their biosecurity protocols [20]. Key control strategies include physical fencing, population control (using trained detection dogs and drone teams), efficient surveillance, and multi-sectoral coordination.

For wild boar management, key strategies include actively searching for and safely disposing of carcasses (a major virus reservoir), reducing population density through hunting, and using fencing to restrict movement. Research findings and field experience from ASF-affected countries in the EU indicate that implementing fencing, combined with other control measures such as culling diseased pigs and removing carcasses, can effectively reduce wild boar movement and help contain the spread of ASF in wild boar populations (EFSA, 2024; https://efsa.onlinelibrary.wiley.com/doi/10.2903/j.efsa.2024.9095). A 2024 review systematically assessed the effectiveness of fencing and other population-segregation methods, drawing on both scientific studies and questionnaire-based field experiences. The review found that fencing can reduce wild boar movement, but its effectiveness is influenced by multiple factors, including fence characteristics, landscape features, and timing of implementation (EFSA, 2024). The effectiveness of hunting is debated, showing limited short-term impact but potential long-term benefits in reducing infection prevalence [97]. Prompt removal of carcasses near pig farms has been shown to significantly reduce infection risks in domestic herds [98]. The Czech Republic combined fencing with intensive hunting, cutting wild boar density by up to 50% in some areas in the short-term (European Commission Directorate-General for Health and Food Safety, 2025; https://ec.europa.eu/food/audits-analysis/audit-report/download/17092). Belgium adopted fencing, forest closures, intensive search-and-destroy campaigns, hunting, and trapping. Every sick pig must be tested for ASF regardless of symptoms; over 22,000 samples have been taken, and the number is still rising (EuroMeat News.com). In Saxony, Germany, hunters worked with forestry and government agencies, backed by 35 specially trained cadaver dog teams and drone-assisted searches (Food and AgriBusiness News). Spain has rolled out comprehensive control and eradication measures, including large-scale carcass searches, physical barriers and fencing, “white zones” to lower wild boar density, and stringent biosecurity on pig farms, efforts that have so far effectively contained viral spread (WOAH, 2026).

In November 2025, ASF re-emerged in wild boar in the Catalonia region of Spain after more than 30 years of absence. The origin of the virus remains unknown, although investigations have ruled out a laboratory escape. After five and a half years of control, Saxony declared ASF eradication on 5 February 2026, but lost its disease-free status less than two months later when infected wild boar were found in Görlitz (Saxon State Ministry for Social Affairs, Health and Social Cohesion). This episode underscores ASF’s unpredictability: even after a year without cases, the threat persists. Strengthening farm-gate biosecurity and restricting unauthorized access remain the industry’s best defenses (Swineweb, 2026; https://swineweb.com/asf-resurges-in-germanys-saxony-region-bio-security-focus-returns-to-human-vectors/). During outbreaks, official control measures typically involve establishing restriction zones (e.g., protection and surveillance zones) around infected premises, enforcing standstills, and conducting thorough cleaning and disinfection. However, ASF control in smallholder or backyard farming systems presents distinct challenges that extend beyond these standard interventions. These farms are often widely dispersed, engage in numerous informal trading points, and are embedded within complex social networks influenced by factors such as poverty and ethnic practices. Consequently, in addition to the measures applied in intensive commercial operations, effective control requires targeted public education and awareness campaigns to improve understanding of the disease and promote reporting. Where feasible, providing appropriate government subsidies to compensate farmers for the proper disposal of diseased or dead pigs may also serve as a critical incentive, helping to reduce the risk of ASF transmission within smallholder settings.

### Comparison of control models across countries and regions

**Success stories in eradication:** The Czech Republic and Belgium successfully eliminated ASF from their territories through rapid and rigorous measures focused on wild boar. This included intensive passive surveillance (carcass searching and removal), population reduction in the core area, and the construction of physical barriers to prevent wild boar movement [28]. The Czech Republic (2017–2019) was the first EU country to eradicate ASF and regain disease-free status. The first case was detected in a wild boar carcass in the Zlín region in June 2017, and by December 2018, a total of 230 wild boar cases had been confirmed, while domestic pig farms remained unaffected throughout. During the outbreak period, a total of 29,381 wild boar samples were tested by PCR and 14,227 by serology; for domestic pigs, 2,717 PCR tests and 2,324 serological tests were performed. The European Commission recognized the country as ASF-free on 12 March 2019 by abolishing regionalization, and the WOAH confirmed the resumption of self-declaration on 19 April 2019 (SANTE Data Collection Platform). Belgium (2018–2020) reported its first ASF detection in wild boar in September 2018. Control measures focused on eradication successfully confined the virus to the affected area, and domestic pigs remained infection-free. A total of 5,422 wild boar were examined, of which 833 tested positive. The last positive fresh wild boar carcass was recorded on 11 August 2019, and no new cases were detected over the subsequent year of surveillance. The current estimated cost of wild boar management measures is approximately €300/ha [99]. The WOAH confirmed Belgium’s return to ASF-free status in December 2020 (EuroMeat News.com). Saxony (2020–2025) recorded its first case on 31 October 2020 in a wild boar near the Polish border. Over the entire period, a cumulative total of 2,398 wild boar cases were confirmed, with no infections in domestic pig farms. After five and a half years of intensive control, Saxony declared ASF eradication on 5 February 2026. During the control effort, approximately 830 km of protective fencing was installed, of which 480 km has since been dismantled; the total cost amounted to around €60 million. However, the state has now lost its disease-free status following new detections (EuroMeat News; Tridge News). Sardinia, Italy, after decades of endemicity, is on the verge of eradication. Its success is attributed to a comprehensive strategy that banned free-range pig production, strengthened biosecurity on outdoor farms, and provided government subsidies for compliance [87].

**Challenges in long-term control:** In many European countries (e.g., Poland, Germany) and across Asia, ASF has become persistent. Key challenges include the vast territories with self-sustaining virus cycles in resilient wild boar populations, the high density of small-scale farms with low biosecurity, and gaps in the implementation of control measures due to logistical or social constraints [29]. Human activities, such as the long-distance illegal movement of animals and contaminated products, continue to drive virus spread. The case of Poland demonstrates that even with a decline in domestic pig outbreaks, the wild boar reservoir remains a constant threat, requiring sustained, coordinated efforts between veterinary services, hunters, and farmers.

## VACCINE DEVELOPMENT PROGRESS AND PRACTICAL DILEMMAS

Inactivated virus vaccines have proven ineffective, consistently failing to induce protective immunity against ASFV infection [100]. In contrast, LAVs represent the most advanced candidates. These include naturally attenuated strains and genetically modified viruses with targeted deletions of virulence genes, such as the I177L deletion mutant, which has become commercially available in Vietnam [101].

The development of ASF vaccines, particularly LAVs targeting the globally prevalent highly virulent genotype II virus, has now entered an unprecedented phase of field validation. Vietnam pioneered this effort, becoming the first country to approve and commercialize three genotype II-based LAVs between 2022 and 2023: Navetco vaccine (ASFV-G-ΔI177L), AVAC vaccine (ASFV-ΔMGF), and Dabaco vaccine (ASFV-G-ΔI177L/ΔLVR). All three have received circulation certificates under Vietnam’s Animal Health Law and technical standards, are indicated for pigs from 4 weeks of age, and are effective against genotype II ASFV strains. These approvals mark a global shift in ASF control strategies, from sole reliance on biosecurity and culling to a new phase of integrated “vaccine-plus-comprehensive” approaches (Nongnghiepmoitruong, 2025; https://van.nongnghiepmoitruong.vn/detailed-instructions-on-asf-vaccination-are-soon-to-be-issued-d767433.html). Among these, AVAC live vaccines has been monitored across 610 farms and proven safe and effective, achieving 100% antibody response rates in provinces such as Lang Son, Cao Bang, and Quang Ngai. Field reports indicate a single dose provides up to 90% protective efficacy, with immunity lasting 4–5 months (Nongnghiepmoitruong, 2025). An independent study further confirmed that vaccinated pigs maintained 100% protection against genotype II challenge at both 28 and 150 days post-vaccination [41]. In December 2025, the Philippine government reported 90% vaccine efficacy in its ASF vaccination pilot, which covered nearly 500,000 hogs across 12 semi-commercial farms, 9 smallholder farms, and 1 government-run farm, using AVAC live vaccines. However, the rollout remains under government pilot control and has not yet entered large-scale commercial use (Philippine News Agency).

Live-attenuated ASF vaccines offer substantial benefits, including high protective efficacy, durable immunity, a strong safety profile (with no evidence of adverse effects in breeding sows or horizontal/vertical transmission), and demonstrated disease control in vaccinated areas. While some LAVs confer strong protection against homologous strains, their deployment is hindered by significant hurdles. These include safety concerns regarding residual virulence, adverse reactions, and the potential for recovered animals to become chronic carriers. Furthermore, the lack of a DIVA (Differentiating Infected from Vaccinated Animals) strategy severely complicates surveillance and eradication efforts. Compounding these issues is the limited cross-protection afforded by current LAVs against the diverse ASFV genotypes circulating globally, alongside substantial production challenges, as manufacturing relies on primary pig macrophages and lacks a scalable, continuous cell line [102]. In light of this, the FAO White Paper (2025) recommends that ASF vaccination be deployed only within the framework of well-established official control programs, sustained surveillance, and robust veterinary oversight, while WOAH emphasizes that any vaccination strategy must be grounded in thorough risk assessment and local epidemiological characteristics.

Novel vaccine approaches, such as subunit, DNA, and viral-vector vaccines, are under investigation but have not yet achieved the protective efficacy of LAVs in challenge studies [10]. Illegal vaccines, defined as unlicensed products with unverifiable sources and quality, pose risks that, in the absence of robust regulation, far outweigh any conceivable benefits. WOAH has repeatedly emphasized that the use of substandard vaccines is counterproductive, potentially leading to virus dissemination and recombination, and therefore urges member countries to employ only high-quality, officially approved vaccines (WOAH, 2025). A major emerging concern is the appearance of natural recombinant viruses (genotype I/II), which may evade immunity conferred by current genotype II-based vaccine candidates, underscoring the need for ongoing research and versatile vaccine strategies. None of the three genotype II LAVs currently commercialized in Vietnam confer effective cross-protection against recombinant genotype I/II ASFV strains [103]. Nevertheless, a successful and safe vaccine remains a potential game-changer in the long-term control of ASF, particularly for protecting the global commercial swine herd. Through emerging technologies and sustained international collaboration, future ASF LAVs should embody three critical attributes: (1) broad efficacy, providing cross-protection against circulating and recombinant genotypes; (2) biosafety, achieved through conditional replication restriction or precise attenuation; and (3) DIVA compatibility, paired with a diagnostic framework to support post-vaccination monitoring [104].

In China, the Harbin Veterinary Research Institute of the Chinese Academy of Agricultural Sciences achieved a milestone in May 2019 with its self-developed ASF vaccine candidate [105]. By early 2026, ASF cases in the Philippines had dropped dramatically, indicating notable success in disease control. The government-led vaccination campaign is considered a key factor in this progress, although the vaccine has not yet been widely commercialized (Philippine Information Agency). WOAH continues to urge all countries with vaccination programs to share relevant information. In October 2025, the FAO published a white paper on ASF vaccination, providing a comprehensive policy-consideration framework for competent authorities and the livestock industry. The EU-funded VACDIVA project is set to deliver three safe and effective pilot vaccines for wild boar and domestic pigs, now ready for registration, while also validating diagnostic assays, developing cost-effective surveillance and vaccination strategies, and conducting field trials in Kenya and Lithuania, with epidemiological modeling services to support global disease control (European Commission, A safe DIVA vaccine for African Swine Fever control and eradication). In May 2025, WOAH adopted the first international standards for ASF vaccine production, establishing core requirements for safety, efficacy, and matching circulating genotypes; in May 2026, WOAH issued the first global guidelines for field evaluation and post-vaccination monitoring, offering standardized tools for generating real-world evidence and emphasizing transparency, stakeholder collaboration, and surveillance for virulence reversion and recombination risks. Collectively, these initiatives mark a significant shift from fragmented national approaches to internationally coordinated ASF vaccine policies.

## DISCUSSION AND FUTURE PERSPECTIVES

This review synthesizes evidence demonstrating that ASF has solidified its status as a global pandemic, sustained by complex transmission cycles involving wild boar, driven by human activities, and complicated by the continuous evolution of the virus, particularly the emergence of the dominant genotype II and, more recently, concerning inter-genotypic recombinants. Despite nearly a century of knowledge, significant gaps remain, and the persistence of the virus in wild boar reservoirs, as seen in Europe, renders eradication in affected regions a long-term challenge [80].

Looking ahead, a multifaceted and globally coordinated approach is paramount. ASF has entered a new phase defined by a persistent wild-boar reservoir, the emergence of recombinant strains, and vaccine deployment. Although global domestic pig outbreaks have declined since September 2025, wild boar cases have continued to rise in parts of Europe and Asia during the same period, underscoring that the persistent wildlife reservoir remains a major obstacle to eradication. Recent WOAH data offer cautious optimism, with slowing domestic spread in some regions; however, sustained circulation in wild boar demands a continued One Health approach that integrates wildlife, domestic pig, and environmental management. The possible final outcomes and their probabilistic pathways may vary across regions, depending on the divergence of global epidemic dynamics and differences in control capacities (Table 2).

**Table 2:** African swine fever endgame scenarios: characteristics, target regions, and probable pathways.

**Endgame Scenario**	**Key Characteristics**	**Applicable Regions**	**Probable Pathway**
Eradication	Virus eliminated in both wild boar and domestic pigs; restoration of ASF-free status	Geographically isolated areas with low wild boar density and strong governance capacity	Early detection + perimeter fencing + intensive hunting + carcass removal + strict border control
Endemic Stability	Low-level viral circulation in wild boar populations; domestic pigs remain disease-free via biosecurity	Continuous wild boar habitats, moderate governance capacity, high proportion of smallholder farms	Long-term monitoring + high-risk zone management + wild boar population regulation + vaccine buffering
Vaccine-dependent Control	Epidemics controlled in domestic pigs through mass vaccination; wild boar remain as viral reservoirs	Dense domestic pig populations, intractable wild boar populations, vaccine production capacity	Commercial vaccines + regular booster immunization + DIVA surveillance + wild boar prevention and control

The future of ASF surveillance lies in integrating advanced technologies that can detect and predict outbreak risks with greater precision. As revealed by the spatiotemporal models discussed, ASF spread exhibits predictable patterns that can be effectively leveraged by AI-driven early warning systems. The value of such approaches has been demonstrated through initiatives like the ASF modeling challenge, which highlighted the utility of comparative epidemiological modeling for understanding transmission dynamics and evaluating the potential impact of different control measures [56]. Furthermore, innovative and cost-effective approaches are emerging, such as the monitoring of online animal price data as a novel early warning signal for impending disease outbreaks, an approach that has shown considerable promise in preliminary studies [106, 107]. Collectively, these technological advancements offer new opportunities to move from reactive outbreak control toward proactive risk prediction and prevention. Meanwhile, the rapidly shifting global dynamics underscore the urgent need for harmonized reporting standards. WOAH should continue to encourage member countries to share epidemiological and genomic sequencing data in a timely and transparent manner.

Addressing the complex challenge of ASF requires a holistic approach integrating veterinary science, ecology, social sciences, and data science. A comprehensive understanding of the disease and the development of effective mitigation strategies require not only insights into viral biology and host immunity but also a deep comprehension of the human behaviors, economic drivers, and ecological interactions that facilitate viral transmission and persistence. Given the transboundary nature of ASF, robust global coordination mechanisms are essential. Effective prevention and response depend on standardized reporting protocols, harmonized control policies across national borders, and the systematic sharing of resources, data, and knowledge to enable rapid and efficient action against incursions. While strict biosecurity remains the primary and most reliable method for preventing the introduction and spread of ASF [108], the pursuit of a safe and effective vaccine must continue with urgency. Parallel research into antiviral drugs and optimized virus inactivation strategies also holds promise for broadening the arsenal of available control tools [101]. Future research should address critical knowledge gaps by quantifying the role of recovered or chronically infected pigs in long-term virus maintenance, assessing ASFV persistence in soil, water, and under different pH conditions and its contribution to transmission, and establishing a global surveillance network to monitor the spatiotemporal evolution of recombinant strains [109, 110].

Although ASF is not a zoonotic disease, its impacts resonate profoundly within a One Health framework. The virus causes significant disruption to ecosystems, evidenced by population declines in affected wild boar. It threatens global food security by destabilizing pork production, a critical protein source for billions of people. Furthermore, it inflicts profound socio-economic losses on rural communities and national economies, undermining livelihoods and trade. Consequently, controlling ASF is not merely an animal health issue but a critical component of ensuring ecological balance, economic stability, and the sustainability of global food systems.

In light of the epidemiological characteristics of ASF, a concerted global effort that combines enhanced surveillance, interdisciplinary research, and robust international cooperation is essential to mitigate the impact of this devastating disease and safeguard pig production worldwide. Future modeling and predictive efforts should incorporate a broader range of anthropogenic and social factors, including human population density, the location of live animal markets and trading points, road network density, educational attainment levels, transportation routes, and the distribution of pork processing facilities. By integrating these variables, epidemiological models can achieve greater predictive accuracy, thereby enabling veterinary authorities to respond more rapidly and deploy resources more effectively in the event of an outbreak. In conclusion, while the challenges posed by ASF remain substantial and enduring, there are reasons for cautious optimism. Persistent innovation, unwavering political will, and sustained investment in research and control infrastructure offer genuine pathways toward containing this disease.

## CONCLUSION

This review synthesizes the global epidemiology of ASF, demonstrating its transformation into a persistent pandemic driven by complex transmission cycles involving wild boar reservoirs, human-mediated long-distance spread, and ongoing viral evolution, particularly the dominance of genotype II and the emergence of recombinant and vaccine-like strains. Key findings highlight a post-2025 tipping point: declining domestic pig outbreaks globally contrasted with rising or persistent wild boar cases across Europe and Asia, underscoring the wildlife reservoir as the primary barrier to eradication.

**Effective control requires region-specific strategies:** rigorous biosecurity and wild boar management in Europe, integrated vaccination-plus-biosecurity programs in Asia, and strengthened smallholder support with economic incentives in Africa. The cautious success of genotype II LAVs in Vietnam and the Philippines demonstrates the potential of vaccination when implemented under official oversight, but also warns against unregulated use, which may accelerate viral evolution.

This review integrates post-2025 data, provides a balanced One Health perspective, and synthesizes recent developments in recombinants, vaccine deployment, and spatiotemporal modeling.

As a single-author narrative review relying primarily on English-language sources, it may have missed some non-English literature. The rapidly evolving nature of the ASF situation means new developments after June 2026 are not captured.

Priority research should focus on broad-spectrum vaccines that offer cross-protection against recombinants, quantification of chronic carrier roles, environmental persistence studies, and the development of AI-augmented predictive systems that integrate genomic, mobility, and socio-economic data. Establishing a truly global genomic surveillance network and harmonized reporting standards will be essential.

In conclusion, while ASF remains a formidable challenge, persistent innovation, strengthened international coordination, and sustained investment in research and control infrastructure provide genuine pathways toward long-term containment and eventual eradication. A unified One Health approach integrating enhanced surveillance, adaptive vaccination, and robust biosecurity offers the best opportunity to safeguard global swine production and food security.

## DATA AVAILABILITY

The supplementary data can be made available from the corresponding author upon request.

## AUTHORS’ CONTRIBUTIONS

JB: Conceptualized and designed the review, conducted the literature search, interpreted the evidence, drafted the manuscript, and critically revised the manuscript. The author has read and approved the final manuscript.
